# A temporary hospital intensive care unit: a preparedness concept to increase intensive care capacity

**DOI:** 10.1186/s13049-024-01301-2

**Published:** 2024-12-05

**Authors:** Øyvind Østerås, Stian Almeland, Håvard Landsdalen, Stig Gjerde, Jan Knudtzon Sommerfelt-Pettersen, Hans Flaatten

**Affiliations:** 1 Department of Anaesthesia and Intensive Care, Haukeland University Hospital, Bergen, Norway; 2 Department of Clinical Medicine, Faculty of Medicine, University of Bergen, Bergen, Norway; 3 Norwegian National Burn Center, Department of Plastic, Hand, and Reconstructive Surgery, Haukeland University Hospital, Bergen, Norway; 4 Haukeland University Hospital, Bergen, Norway; 5 Department of Research and Development, Haukeland University Hospital, Bergen, Norway; 6 Department of Clinical Medicine, University of Bergen, PO Box 7804, Bergen, 5020 Norway

**Keywords:** Critical care, Intensive care unit, Emergency preparedness, COVID-19

## Abstract

**Introduction:**

Norway faced the possibility of the most significant strain on its intensive care capacity in decades during the COVID-19 pandemic. All Regional Health Authorities in Norway were instructed to prepare for an increase in Intensive Care Units (ICU) capacity demands. To address the surge in demand for critical care, a gymnasium within Haukeland University Hospital premises was planned to be used as a 20-bed temporary ICU. A team-based care approach was trained, where non-ICU nurses received specialized training to support ICU procedures. Maintaining up-to-date medical devices and consumables stored for preparedness through a planned rotation system that feeds into daily use are important. While shortages of medical equipment, hospital beds, and intensive care facilities may occur, personnel shortages are likely to be more significant.

**Conclusions:**

The concept demonstrates promising potential in enhancing preparedness and maintaining critical care surge capacity during pandemics or mass casualty incidents.

## Introduction

When the COVID-19 pandemic struck Europe in February 2020, critical care capacity in many European countries was quickly overwhelmed. Emergency preparedness plans cannot anticipate and address every possible extreme scenario. However, the Nordic countries, including Norway, have consistently maintained a low ratio of critical care beds per inhabitant for many years compared to most European countries. In 2012, Norway had eight critical care beds per 100.000 inhabitants, including both intensive care and intermediate care beds [1]. The disparity between the existing number of intensive care beds and the surge capacity became evident in March 2020 when Norwegian health authorities projected a potential requirement of 1,200 intensive care beds in the country (equivalent to 24 beds per 100,000 inhabitants). Recognizing the magnitude of the challenge, all Regional Health Authorities were instructed to prepare for this increased demand for intensive care capacity [2].

Norway faced the possibility of the most significant strain on its intensive care capacity in decades. Consequently, there was a pressing need for agile solutions to prevent a breakdown of a vital part of the national health service. The urgency to find effective and adaptable solutions became evident as authorities sought to avert a crisis and ensure the provision of critical care services to those in need. The aim of the study was to present the preparedness concept of a 20-bed temporary ICU (TICU).

### Haukeland university hospital

Haukeland University Hospital is the largest hospital within the Western Norway Regional Health Authority. It serves as a general hospital for 468,000 inhabitants and a regional hospital for 1.14 million inhabitants across an area of around 43,000 km^2^ in Western Norway [3]. Currently, Haukeland University Hospital operates with a daily capacity of eleven beds in the general intensive care unit (ICU), along with nine beds in other specialized ICUs (cardiac ICU and cardio-thoracic surgical ICU). Additionally, the Children and Youth Clinic has four neonatal ICU beds, and three intensive care beds are allocated to the Norwegian National Burn Center. However, to meet the projected demand for ICU beds set by the Norwegian health authorities in March 2020, Haukeland University Hospital would need to increase the number of available intensive care beds by more than fivefold, a substantial increase.

Within Haukeland University Hospital’s pandemic preparedness plan, the general ICU serves as the primary department for critical care for patients affected by a pandemic. The department can isolate patients in three pandemic cohorts, each consisting of five ICU beds if needed. An airborne infection isolation room with two beds and additional single-bed rooms are also available. However, if the number of pandemic patients exceeds 21, any further increase in pandemic admissions requiring intensive care would necessitate using an adjacent post-surgical recovery unit, resulting in a near-complete shut-down of major surgeries within the hospital. Alternatively, dedicated facilities to address pandemic patients’ critical care needs must be identified. This realization prompted an intensive search for solutions to address the worst-case scenario of the pandemic during the final weeks of March 2020. The escalating demand for intensive care capacity forced the search for supplemental facilities within the hospital that could be transformed into critical care units. It became evident that it would necessitate innovative planning to establish and operate an alternative ICU within the hospital. This marked the beginning of the creation of the preparedness concept of a planned temporary ICU, driven by the urgent needs triggered by the pandemic.

### A temporary ICU

**Fig. 1:**
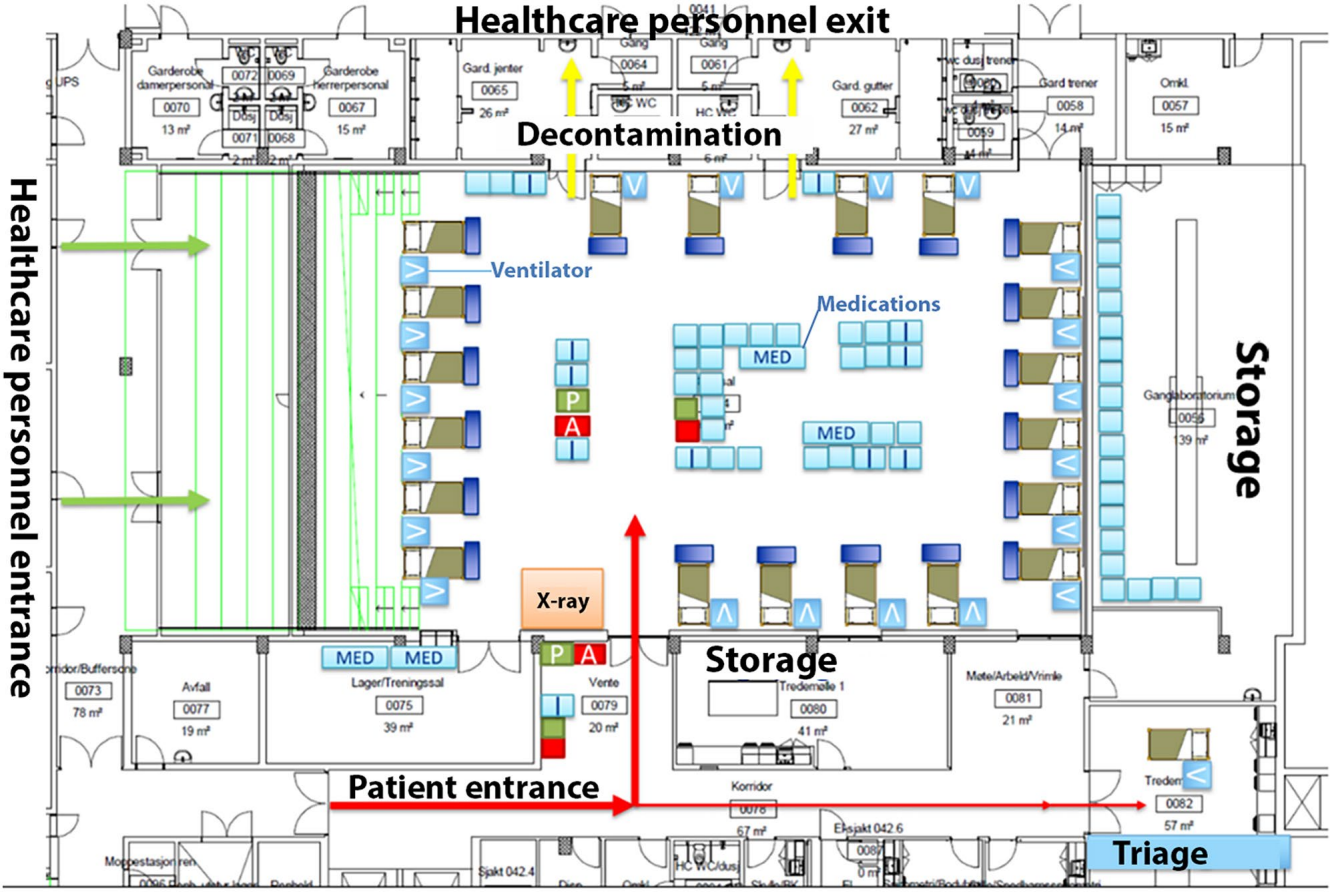
An illustration of the gymnasium, surrounding rooms, hospital beds and medical equipment

The concept was to assemble pandemic patients into a single and large cohort, as they were suffering from the similar viral infection. To achieve this, the idea of utilizing a gymnasium within the hospital, typically designated as a training and play area for hospitalized children, emerged as a viable option for establishing a separate critical care zone. The gymnasium, located in a newly constructed hospital building fully integrated within the hospital facilities, offered sufficient space with its 443 square meter area, allowing for the temporary establishment of 20 ICU beds (Fig. 1). To ensure proper infection control, the gymnasium and surrounding rooms were designated as contagious areas, always necessitating using masks and infection protection suits. The lockers usually utilized for sports activities were supposed to serve as exit points for safely removing protective suits, while other rooms were repurposed as medication rooms, storage facilities, and break rooms. The planned utilization of the available facilities inside and around the gymnasium allowed an organized and effective critical care environment for pandemic patients.

Establishing 20 beds for critically ill patients in a gymnasium posed an unconventional and challenging idea. While ensuring a suitable facility was one of the primary challenges in preparing for potential shortages in ICU capacity, it was important to recognize that there were other significant hurdles to overcome. One advantage of gathering 20 patients in a large cohort would be the ability to operate on a larger scale, which can be achieved with a reduced ICU staff. This approach allowed for the efficient allocation of limited resources and personnel, enabling the medical team to provide comprehensive care to a larger number of patients within the designated area.

### Personnel

**Fig. 2:**
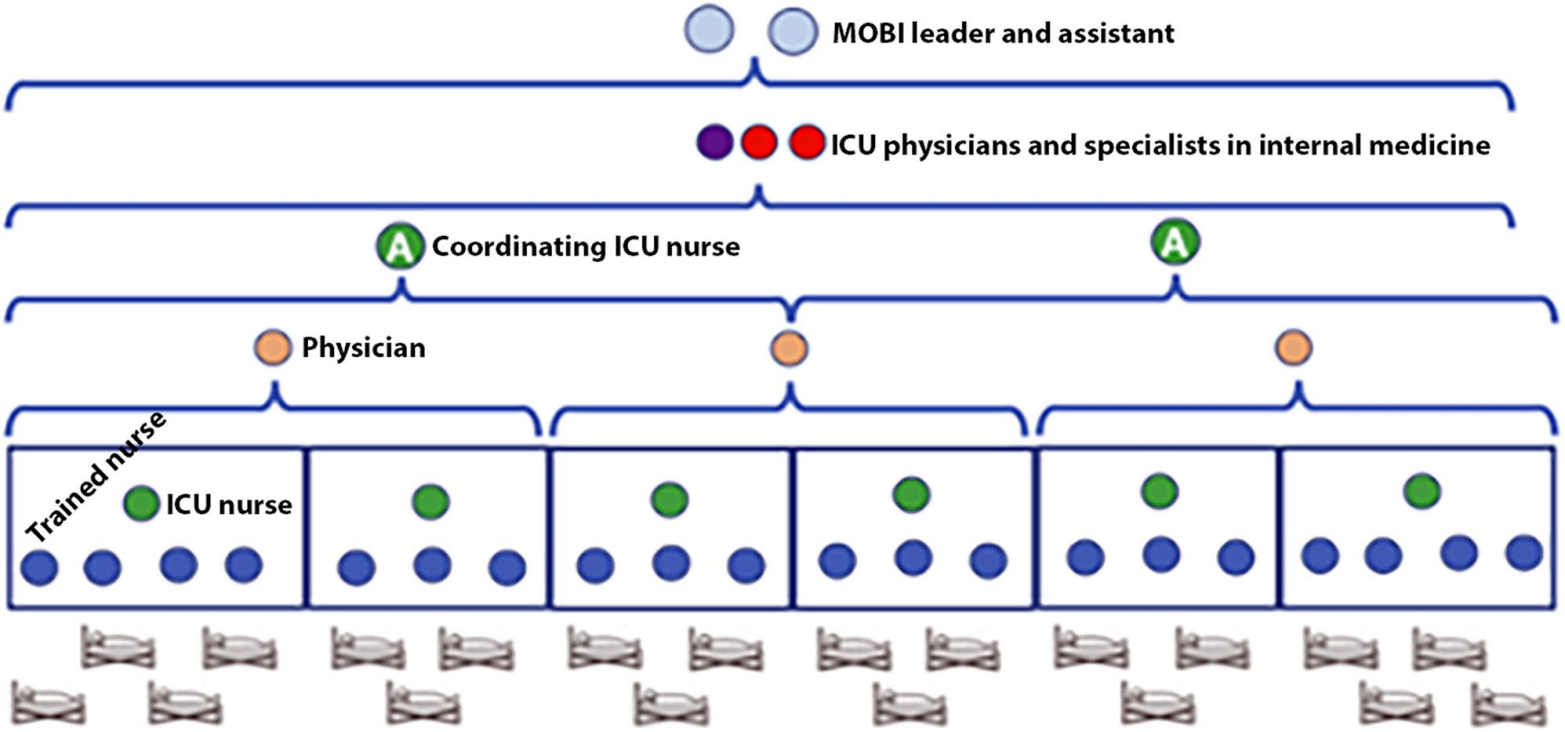
Staffing of the planned temporary ICU. Trained nurses were not daily working in ICU but were trained for working in TICU. Physicians were daily working with anesthesia or specially trained for working in TICU

During normal ICU operations in Norway, the standard approach to intensive care nursing involves a one-nurse-to-one-patient ratio. According to a recent Norwegian report, the recommended physician-patient ratio for intensivists ranges from 1:3 to 1:2 [4]. However, the preparedness plan necessitated a shift towards team-based care. During a pandemic, it is expected that several ICU staff members may be absent due to their own or their family members’ infection. Consequently, it may not be realistic to increase the number of nurses and physicians with intensive care experience to meet the surge in demand. In the planning of TICU, the bedside nurse responsible for patient care was likely to be a registered nurse from another department, working as part of a team of 3–5 nurses led by an intensive care nurse (Fig. 2), following the concept of team nursing. To address the increased need for nursing staff in the ICU, 44 non-ICU nurses completed a two-week national training program, which includes extensive theoretical and practical ICU training tailored explicitly for COVID-19 patients. The objective was to quadruple the non-ICU nursing staff. These nurses were recruited from various wards within the hospital, most of whom had no prior ICU experience. Similar use of team nursing and training programs were used during the COVID-19 pandemic in Italy [5]. Additionally, nurse anesthetists and perioperative nurses have also undergone training to be part of the team. The principle of team care extends to physicians as well. Other anesthesiologists, and potentially physicians from various specialties within the hospital, were to be incorporated into physician teams, under the leadership of intensivists.

### Medical equipment

Two primary principles have been established for medical equipment allocation to the TICU. Firstly, additional medical devices, consumables, and equipment should always be readily stored and accessible for use with the 20 ICU beds. Secondly, the equipment must remain up to date and within the expiration date. To meet this requirement, software-driven medical devices and computers should be regularly rotated with similar products used in daily hospital operations, at least every six months. This rotation ensures that the medical staff remains familiar with the equipment. For this planned concept, medical consumables will be stored in large cabinets equipped with casters to facilitate the rotation process. These cabinets will be rotated twice a year and moved to the storage, with the consumables from the cabinets used during the previous six months replaced with new supplies. By following this procedure, the unit will always have an updated inventory of consumables immediately available for use. The storage of medications for TICU will be addressed by increasing the ICU medication storage capacity to accommodate an additional three months’ worth of regular ICU consumption. This surplus supply will be replaced after use in the everyday operational needs of the ICU.

### Exercises

**Fig. 3:**
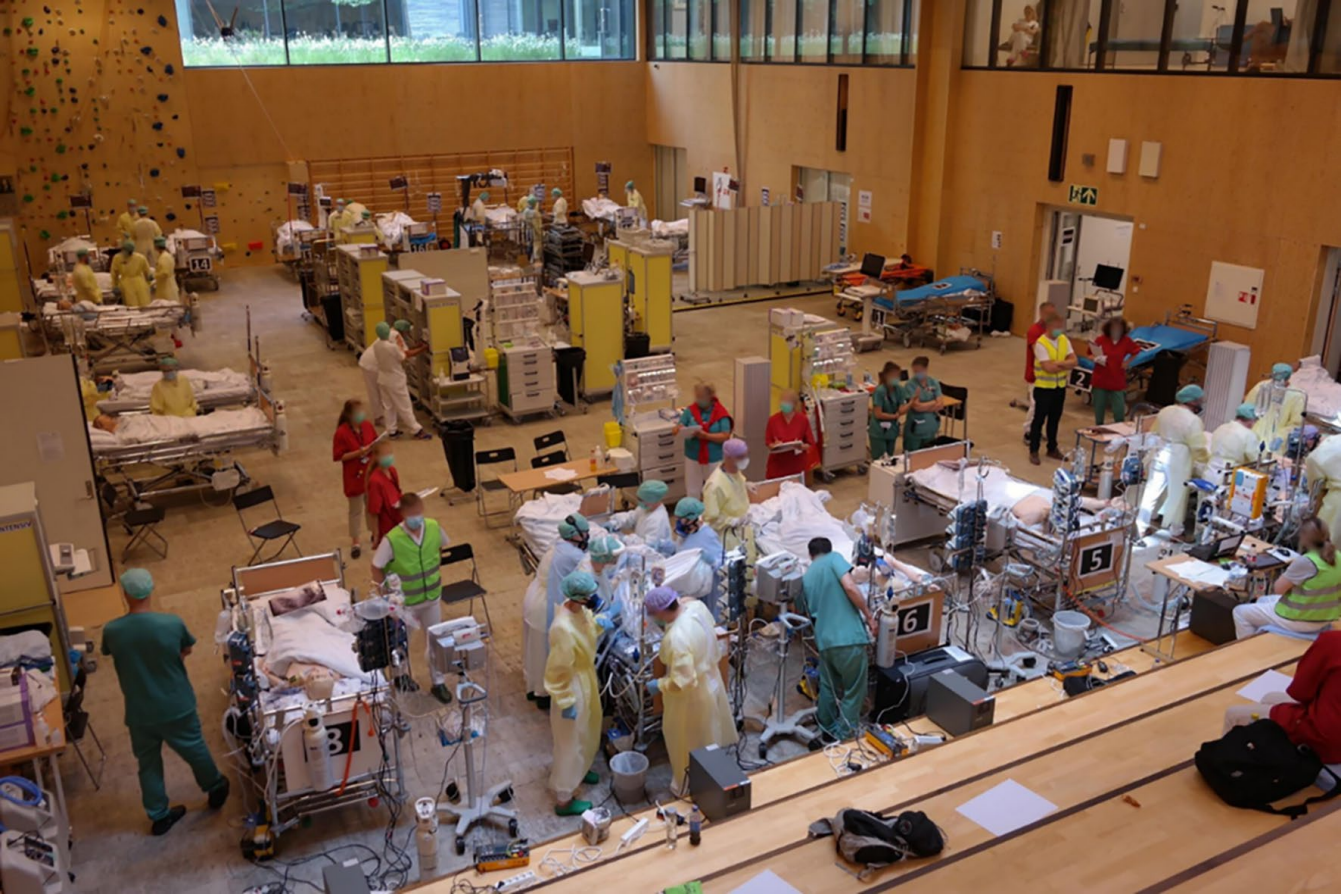
Picture from the gymnasium during an early full-scale exercise

A full-scale exercise was conducted merely three months after the inception of the concept. This exercise involved nearly 60 individuals from Haukeland University Hospital (Fig. 3), incorporating the utilization of manikins and simulated patient cases. The evaluation of this exercise revealed several noteworthy findings. One of the key observations was the potential benefits of implementing structural modifications in the gymnasium. These modifications would entail the installation of fixed pipelines for medical gases, electrical power, and network connections. Furthermore, the exercise highlighted the necessity of procuring additional medical equipment dedicated to TICU preparedness.

The project received full support from hospital management. The gymnasium’s infrastructure was upgraded to accommodate the requirements as part of the project implementation. Specifically, 23 electrical power, oxygen, and medical gas outlets have been installed within the gymnasium’s walls, concealed behind built-in hatches. These hatches can be closed, enabling the gymnasium to be fully utilized between rare establishments of exercises or ICU capacity shortage. To further enhance the functionality of the space, the gymnasium can be divided into two separate cohorts by utilizing a downfolding wall. This partitioning allowed for the segregation of patients or different groups. Movable hospital screens on casters were employed between the beds. These screens acted as barriers, reducing both visibility and noise. While the ventilation system in the gymnasium was relatively new and designed to meet the requirements of sports events, it falls short of the standards needed for an ICU.

The TICU was designed as a distinct section within the Department of Anaesthesia and Intensive Care to ensure efficient maintenance and operation. This separate structure allows seamless integration with the hospital’s electronic patient chart system, enabling bedside ordering of medical equipment, consumables, laboratory tests, and radiology procedures. It also streamlines the process of ensuring accurate delivery of these resources. Additionally, organizing TICU as an easily accessible yet inactive section clarifies the responsibilities for maintenance and operational tasks, providing effective management.

A tabletop exercise was conducted to identify potential risks and vulnerabilities during the initial stages and early days of a possible TICU deployment. In a pre-planned establishment exercise, six fully equipped ICU beds were made available within 45 min. Additionally, a comprehensive full-scale exercise is planned to test the entire preparedness plan, covering all aspects from the initial call out to the dismantling.

## Discussion

TICU is an essential part of the hospital’s preparedness plan. It allows the capacity to establish a fully functional additional ICU within the hospital building. This capability may prove invaluable in situations where there is a shortage of ICU capacity, such as during a pandemic, mass casualty event, or when immediate evacuation from existing ICU facilities is required.

Adopting a more generic approach to the concept, as medical devices and consumables are stored in a centralized depot, greatly enhances the capability to establish TICU in various locations according to specific needs and circumstances. This flexibility ensures the hospital’s readiness to respond effectively to critical situations and offer high-quality intensive care when required. The concept is currently closely associated with the gymnasium in a nearby hospital building. The modifications in the gymnasium enable rapid deployment of TICU, while acquiring dedicated equipment will further enhance the preparedness. However, there are plans to make the concept more adaptable and versatile. The goal is to develop standardized plans and procedures that allow for the establishment of TICU in various facilities, including civilian indoor gymnasiums, large tents, or even ships, provided sufficient electrical power and gas supply.

TICU was not established for patient admissions during the COVID-19 pandemic. A possible future civilian-military cooperation in a national security situation is considered. In the event of a large-scale mass casualty incident, the activation of TICU will likely be considered only when the number of ICU patients reaches a minimum of 20. According to a publication from 2007, the total number of casualties from an accident or terror attack that need to be admitted to the ICU is expected to be approximately 5% [6]. If so, an accident with 400 casualties would provide 20 patients requiring ICU treatment. In such a large incident, Norway can rely on a network of hospitals to provide assistance by establishing an air bridge facilitated by air ambulances, C-130 Hercules military aircraft, and Norwegian Defense contracted Scandinavian Airlines (SAS) medical aircrafts [7].

This concept was improved through three key factors: the dedicated efforts of involved colleagues, the availability of suitable facilities, and the optimal timing. The project was driven by the creativity and innovation of colleagues who played a vital role in its development and progress. The unwavering support from hospital managers proved to be crucial throughout the process. An essential aspect of establishing the concept of TICU relied of the active involvement of colleagues from various hospital departments, emphasizing the need for a shared sense of ownership. Rather than depending on predefined tasks, the challenges at hand were resolved through the collaborative efforts of colleagues who played instrumental roles in shaping the TICU. This approach aligns with the teaming concept, as described by Edmondson, which emphasizes flexibility in tackling independent tasks [8].

Numerous ICUs across the globe are designed with individual patient rooms, mandating a minimum of one-to-one nurse-patient ratio. However, the recent pandemic has underscored the importance of having adaptable patient rooms that can be transformed to accommodate cohorts of four to five beds. Employing team nursing and creating cohorts becomes essential when faced with a significant surge in ICU admissions during a pandemic, as it allows for optimal utilization of available personnel. This concept plays a crucial role in enabling the swift establishment of a large ICU, ensuring a more efficient response to the escalating demands.

To establish TICU in the gymnasium was likely in March 2020 due to the rapidly growing COVID-19 pandemic with uncertain outcomes. This timing led to a push from colleagues involved in the project, making TICU a vital part of the hospital’s preparedness plan. The development of the concept of a TICU represents resilience in health care systems, which is described as the capability to proactively foresee, absorb, and adapt [9]. TICU represents Haukeland University Hospital’s possibility of adapting by increasing the ICU capacity with almost the same number of ICU beds as the total normal-day operational number of ICU beds. However, there are several significant challenges in using TICU during a future pandemic.

The primary hurdle was undeniably the shortage of personnel [9]. This challenge was evident during the COVID-19 pandemic in Italy [5]. The increase in personnel fatigue and sick leave posed a concern both during and after the pandemic. Adopting a team-based care approach ensures the efficient utilization of available healthcare professionals and optimizes patient care, even in the face of limited staffing resources during a pandemic. However, if TICU is to be established for a shorter duration, such as during the management of a mass casualty incident, personnel shortage may not be a significant issue. Another challenge arises when the number of admitted patients exceeds the available beds and rooms, necessitating additional facilities to accommodate them or implementing stricter triage criteria [10]. A third challenge is the potential dilution of intensive care expertise when non-ICU-trained nurses and physicians from other medical specialties are to be brought in to support the bed-side team. With the implementation of the TICU, there is an inevitable reduction in the level of intensive care provided due to this decrease in specialized competence. Consequently, leaders must be mindful that by establishing TICU, they accept a lower ambition in the level of care. During the peak of a pandemic, it is highly likely that care standards and available services will undergo modifications, given the altered circumstances and increased demands on the healthcare system.

Being fully prepared for all kinds of infrequent incidents is an impossible task. However, waiting until a disaster strikes before making plans is an approach that hampers effective response. Therefore, hospitals should proactively develop flexible plans to enhance surge capacity by increasing the number of hospital beds and intensive care beds. Additionally, addressing shortages in essential personnel can be achieved by implementing team nursing and physician teams. By adopting these proactive measures, hospitals can enhance their readiness to respond to unforeseen crises and ensure high-quality critical care. However, it is essential to acknowledge that managing equipment logistics can be demanding. Nonetheless, there is a positive side-effect of having spare equipment stored and readily available, as it enhances the hospital’s overall preparedness for potential delivery issues and delayed shipments of various medical equipment, consumables, and medications.

During the full-scale exercise, participants highlighted the presence of significant noise in the gymnasium. Additional challenges included a limited ventilation and the constrained spacing between intensive care beds, deviating from standard practices. By identifying and resolving these challenges, the overall functionality and effectiveness of TICU can be improved.

Findings and considerations in this study are predominantly based on observations and experience from a single hospital, requiring cautious interpretation.

## Conclusions

The TICU concept demonstrates promising potential in enhancing preparedness and maintaining critical care surge capacity during pandemics or mass casualty incidents. While shortages of medical equipment, hospital beds, and intensive care facilities may occur, personnel shortages are likely to be even more significant. Solutions such as team-based care and specialized training for non-intensive care nurses enable hospitals to allocate resources efficiently and provide essential critical care.

## Data Availability

No datasets were generated or analysed during the current study.

## Abbreviations

COVID-19: Coronavirus Disease 2019 declared a pandemic by the World Health Organization in 2020
ICU: Intensive Care Unit
TICU: A planned temporary hospital Intensive Care Unit
